# Assessment of adverse events via a telephone consultation service for cancer patients receiving ambulatory chemotherapy

**DOI:** 10.1186/s13104-015-1292-8

**Published:** 2015-07-26

**Authors:** Shunsuke Kondo, Satoshi Shiba, Ryoko Udagawa, Yasuaki Ryushima, Miho Yano, Tomoko Uehara, Mihoko Asanabe, Kenji Tamura, Jun Hashimoto

**Affiliations:** Outpatient Treatment Center, National Cancer Center Hospital, 5-1-1 Tsukiji Chuo-ku, Tokyo, 104-0045 Japan

**Keywords:** Telephone consultation service, Ambulatory chemotherapy, CTCAE, Triage

## Abstract

**Background:**

An increasing number of cancer patients are receiving ambulatory chemotherapy to improve their quality of life and reduce medical expenses. During outpatient chemotherapy, adverse events (AEs) occurring at home must be carefully monitored. We investigated the use of our institution’s telephone consultation service that is available to patients and their caregivers for advice on and the management of AEs and complications arising from cancer treatment.

**Patients and methods:**

Telephone consultants assessed and graded AEs according to the Common Terminology Criteria for Adverse Events (CTCAE). All patient characteristics, AEs, and background factors were analyzed using logistic regression analyses.

**Results:**

Between August 2011 and August 2012, we included 253 patients and 344 telephone consultations regarding AEs during chemotherapy for analysis in this study. Grade 1 AEs were assessed in 223 consultations (65%); grade 2 AEs, in 90 consultations (26%); and grade 3 AEs, in 31 consultations (9%). A multivariate logistic regression analysis revealed an association between a change in patient schedules and the occurrence of grade 2 or worse AEs (Hazard ratio = 6.58, *P* < 0.001). Changes in planned chemotherapy occurred more often in cases involving male patients (Hazard ratio = 2.70, *P* = 0.02) and in cases of grade 2 or worse AEs (Hazard ratio = 6.58, *P* < 0.001).

**Conclusion:**

We found that AE assessment using CTCAE via a telephone consultation service is useful for both the triage of patients and the prediction of severe AEs that may change clinical schedules.

**Electronic supplementary material:**

The online version of this article (doi:10.1186/s13104-015-1292-8) contains supplementary material, which is available to authorized users.

## Background

In the oncology setting, there has been an increase in the use of ambulatory treatments, which represent a completely different context to hospitalization. Compared with the inpatient setting, ambulatory chemotherapy results in a better quality of life (QOL) and lower treatment costs for patients. However, administering ambulatory chemotherapy is challenging because of the high volume of patients, time pressures, and the lower level of control. Moreover, patients may need to self-administer essential medications, and side effects often occur at home.

Cancer patients receiving chemotherapy may suffer from treatment-related adverse events (AEs) such as hair loss, nausea, fatigue, and potentially life-threatening effects such as lowered blood counts. Chemotherapy for cancer patients often corresponds with a period of poor QOL because of these AEs; however, most AEs can be managed. Before the initiation of chemotherapy, physicians and nurses talk with patients about potential AEs and the importance of monitoring any changes they notice during treatment to assess toxicity.

Monitoring AEs, including side effects observed during chemotherapy, is a standard part of clinical trials, but should also be a routine consideration in everyday practice because AEs directly influence the QOL of the patient. The Common Terminology Criteria for Adverse Events (CTCAE) is a standard classification system that has been used for reporting AEs in cancer clinical trials [1]. Toxicity data are routinely collected by medical staff; however, both the interpretation and registration of symptoms are susceptible to mistakes, omissions, and misunderstandings due to various factors. The CTCAE includes items derived from measured objective factors, analytical tests, and the patients’ subjective symptoms [2], all of which are currently reported by clinicians. Clinical staff obtain, interpret, and report patient symptoms, a process that can be cumbersome and susceptible to data degradation. In practice, symptoms are abstracted by research support staff members through review of patients’ written medical records.

The telephone has been accepted as a useful means of communication for the management of patient care since the 1960s. Previous research has validated the safety and effectiveness of telephone helplines in many specialist services including depression, pain management, and cancer care [3, 4]. Moreover, telephone services have been shown to be extremely useful to patients because they allow for rapid access to oncology facilities in the event of chemotherapy toxicities [5].

In our hospital, a telephone consultation service for patients receiving ambulatory chemotherapy is managed by the comprehensive care team of the outpatient treatment unit. The purpose of this service is to provide rapid support and assessment of AEs for patients. The use of a telephone service to assess AEs may affect the accuracy of toxicity assessment during ambulatory chemotherapy. Hence, we conducted this study to clarify the potential for predicting severe AEs of ambulatory chemotherapy by patient assessment via a passive telephone consultation service and to investigate the possible benefit of telephone triage using CTCAE.

## Patients and methods

### Survey cohort

The study was conducted in the outpatient treatment unit of the National Cancer Center Hospital, Tokyo. The cases of consultation via the telephone consultation service between August 2011 and August 2012 were enrolled in the study. Any consultation cases regarding issues other than AEs (for example, consultations about the method of dosing or confirmation of the next visiting date) were excluded from the analyses. The study is conducted in accordance with the ‘Helsinki Declaration’, and approved by the institutional review boards of the National Cancer Center Hospital Tokyo (ID: 2012-151). Patients consented to the retrospective use of their medical records for research purposes based on comprehensive permitting system of the National Cancer Center Hospital Tokyo.

### Telephone consultation service

**Fig. 1:**
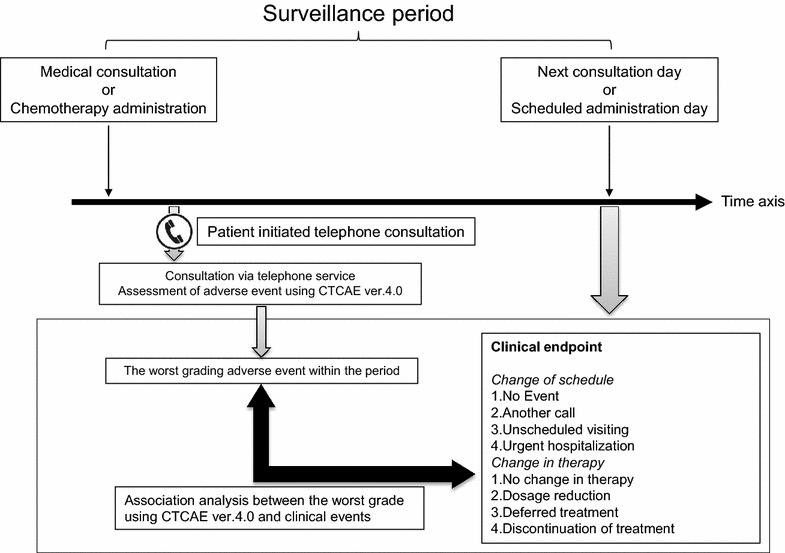
Outline of the telephone consultation service and endpoints of the study.

The telephone consultation service initiated by patients who receiving chemotherapy at the outpatient treatment unit of the hospital was operated by a rotation of staff members consisting of nurses, pharmacists, and medical oncologists of the outpatient treatment unit. Patients receiving chemotherapy in the outpatient setting used the telephone service to talk to a medical professional about all aspects of their chemotherapy, including dealing with side effects, route of administration and their mental health. As part of this service, consultants assessed and graded AEs according to the CTCAE, version 4.0. Consultants referenced common records in the electronic health record system to share patients’ clinical information using the telephone consultation service (Fig. 1).

### Statistical analysis

We set the clinical endpoint of the study as any event that occurred after each telephone consultation in surveillance period. These included changes in clinical schedule before the next scheduled visit (additional consultation call, unscheduled visit, and urgent hospitalization) and any changes in therapy (dosage reduction, deferred treatment, and discontinuation of treatment). We conducted statistical analyses using IBM SPSS version 18.0 (SPSS, Chicago, IL). All patient characteristics and background factors were analyzed using logistic regression analyses. Multivariate logistic regression analyses after univariate analyses were used to reveal any factors that had a strong association with clinical events. *P* values less than 0.05 in a 2-sided test were considered significant.

## Results

### Characteristics of the study cohort

**Fig. 2:**
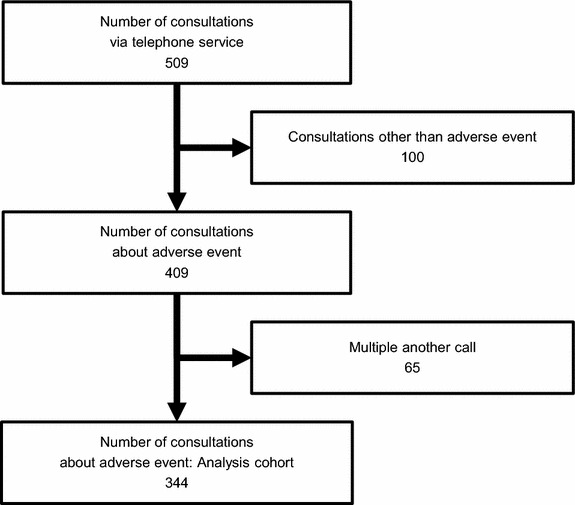
Diagrammatic representation of the study.

In the outpatient treatment center, a total of 25,000 patients were received chemotherapy as outpatients from August 2011 through August 2012. Two-hundred fifty three cancer patients used the telephone consultation service in this same time period. Of the 509 telephone consultations for these 253 patients, 409 consultations concerned AEs during chemotherapy and 65 consultations were excluded because they were repeat calls in a term of surveillance (Fig. 1), resulting in a total of 344 consultations being included in this study (Fig. 2). The 344 subject consultations included in the analyses are described in Table 1. The mean age of patients was 57.2 years (standard deviation, 12.8 years), and 80.5% were female. Most telephone calls were received directly from patients (91.6%), with the remainder of calls made by a caregiver (8.4%). Grade 1 AEs were assessed in 223 consultations (65%), grade 2 AEs in 90 consultations (26%), and grade 3 AEs in 31 consultations (9%). Of the grade 3 AEs, 13 consultations regarding suspicions of febrile neutropenia were included. The most common AEs consulted for were the occurrence of pain (14%), fever (11%), nausea/vomiting (7%), signs of febrile neutropenia (7%), and diarrhea (6%) (Additional file 1: Figure S1A). Chemotherapy regimens of patients included adriamycin + cyclophosphamid (22%), carboplatin + paclitaxel (9%), gemcitabine (7%), S-1 (7%), cyclophosphamide + epirubicine + 5-FU (7%), paclitaxel (7%), and adriamycin (4%) (Additional file 1: Figure S1B).

**Table 1:** Demographic characteristics of the study cohort

Characteristics	Number	%
All grades	344 grades	100.0
Age, years (average, standard deviation)	(57.2, 12.8)	
≤39	33	9.6
40–49	78	22.7
50–59	67	19.5
60–69	94	27.3
70–79	68	19.8
≥80	4	1.1
Sex		
Male	67	19.5
Female	277	80.5
Consulter		
Patients	315	91.6
Caregiver	29	8.4
Cancer type		
Breast cancer	188	54.7
Pancreatic cancer	46	13.4
Ovarian cancer	29	8.4
Colorectal cancer	27	7.8
Soft tissue sarcoma	13	3.8
Gastric cancer	10	2.9
Biliary tract cancer	8	2.3
Liver cancer	7	2.0
Malignant lymphoma	6	1.7
Primary unknown cancer	5	1.5
Other malignancies	5	1.5
Objective of therapy		
Adjuvant chemotherapy	197	57.3
Palliative chemotherapy	141	41.0
Chemotherapy for blood malignancy	6	1.7
Grading of adverse event		
Grade 1	223	64.8
Grade 2	90	26.2
Grade 3	31	9.0

### Association of adverse events and clinical events

For assessment of grade 1 AEs, 184 consultations (83%) led to no change of clinical schedules but of the other 39 consultations (17%), 21 led to another call before the next appointment to see a doctor, 15 lead to an unscheduled visit, and 3 resulted in urgent hospitalization due to AEs. A total of 202 consultations (91%) resulted in no change in therapy, whereas 21 consultations (9%) did result in a change in therapy (13 cases: dosage reduction; 4 cases: deferred treatment; 4 cases: discontinuation of treatment). For assessment of grade 2 AEs, 35 consultations (39%) did not and 51 consultations (61%) did result in a change of clinical schedules (19 cases: additional call before next appointment to see a doctor; 25 cases: unscheduled visits; 11 cases: urgent hospitalization due to AEs). Fifty-three of these consultations (59%) resulted in no change in therapy and 37 consultations (41%) did result in a change in therapy (21 cases: dosage reduction; 5 cases: deferred treatment; 11 cases: discontinuation of treatment). For assessment of grade 3 AEs, 15 consultations (48%) led to no change of clinical schedules and 16 consultations (62%) did result in a change of clinical schedules (6 cases: additional call before next appointment to see a doctor; 2 cases: unscheduled visits; 8 cases: urgent hospitalization due to AEs). Nineteen consultations (61%) resulted in no change in therapy, but 12 consultations (39%) did (4 cases: dosage reduction; 5 cases: deferred treatment; 3 cases: discontinuation of treatment) (Table 2).

**Table 2:** Change in schedules or planned chemotherapy among patients with grade 1–3 adverse events

	Grade 1				Grade 2				Grade 3			
	Adjuvant chemotherapy	Palliative chemotherapy	Blood malignancy	Total	Adjuvant chemotherapy	Palliative chemotherapy	Blood malignancy	Total	Adjuvant chemotherapy	Palliative chemotherapy	Blood malignancy	Total
Event 1: change of schedule (number)												
No event	108	74	2	184	19	16	0	35	11	4	0	15
Another call	19	1	1	21	9	10	0	19	4	2	0	6
Unscheduled visiting	8	5	2	15	12	13	0	25	0	2	0	2
Urgent hospitalization	1	2	0	3	3	8	0	11	2	5	1	8
Event 2: change in therapy (*N*)												
No change in therapy	126	71	5	202	31	22	0	53	12	7	0	19
Dosage reduction	6	7	0	13	8	13	0	21	2	2	0	4
Deferred treatment	2	2	0	4	3	2	0	5	3	2	0	5
Discontinuation of treatment	2	2	0	4	1	10	0	11	0	2	1	3

### Factors associated with clinical events

The results of the univariate analysis revealed a considerable impact of AE grade on changes in clinical schedule (Table 3). The results of the univariate analysis regarding associations with changes in planned chemotherapy are also shown in Table 3. Using the factors from the univariate analysis that were significantly associated with changes in schedule, we then performed a multivariate logistic regression analysis. The results indicated that a change of schedule during ambulatory chemotherapy tended to occur more often with grade ≥2 AEs (Hazard ratio = 6.58, *P* < 0.001). In addition, changes in planned chemotherapy tended to occur more often amongst male patients (Hazard ratio = 2.70, *P* = 0.02) and more often in cases of grade ≥2 AEs (Hazard ratio = 6.58, *P* < 0.001). Although we conducted additional analyses including all variables, these results were materially unchanged.

**Table 3:** Factors associated with clinical events

Characteristics	Event 1: change of schedule			Event 2: change in therapy		
All grades	Hazard ratio	95% CI	*P*	Hazard ratio	95% CI	*P*
Univariate analyses						
Age						
≤64 vs. ≥65	1.08	0.66–1.73	0.77	0.66	0.39–1.13	0.13
Sex						
Male vs. female	1.71	0.99–2.96	>0.05	3.07	1.70–5.52	<0.001
Consulter						
Patients vs. caregiver	0.47	0.22–1.01	>0.05	0.38	0.17–0.84	<0.05
Cancer type						
Breast cancer	0.85	0.54–1.33	0.47	0.55	0.32–0.93	0.03
Pancreatic cancer	1.44	0.76–2.73	0.27	2.14	1.08–4.23	0.03
Ovarian cancer	0.84	0.36–1.97	0.69	0.63	0.21–1.88	0.41
Colorectal cancer	0.56	0.22–1.41	0.22	0.84	0.31–2.29	0.73
Soft tissue sarcoma	1.87	0.61–5.70	0.27	1.18	0.32–4.42	0.80
Gastric cancer	0.91	0.23–3.59	0.89	2.71	0.74–9.87	0.13
Biliary tract cancer	1.28	0.30–5.47	0.74	1.31	0.26–6.65	0.74
Liver cancer	0.85	0.16–4.44	0.85	3.02	0.66–13.83	0.15
Malignant lymphoma	4.34	0.79–24.27	0.09	0.78	0.09–6.78	0.82
Primary unknown cancer	0.53	0.06–4.78	0.57	0.98	0.11–8.89	0.98
Endometrial cancer	1.43	0.24–8.67	0.70	2.66	0.44–16.22	0.29
Objective of therapy						
Adjuvant chemotherapy	0.70	0.51–0.95	<0.05	0.45	0.31–0.65	<0.001
Palliative chemotherapy	1.05	0.84–1.33	0.65	1.57	1.20–2.05	0.001
Chemotherapy for blood malignancy	0.23	0.04–1.27	0.09	1.28	0.14–11.16	0.82
Grading of adverse event						
Grade 2 + grade 3 vs. grade 1	6.70	4.06–11.05	<0.001	6.55	3.67–11.67	<0.001
Grade 3 vs. grade 1 + grade 2	0.38	0.18–0.78	<0.01	0.33	0.15–0.70	<0.01
Multivariate analyses						
Sex						
Male vs. female				2.70	1.15–6.30	0.02
Consulter						
Patients vs. caregiver				1.65	0.67–4.06	0.28
Cancer type						
Breast cancer				0.80	0.36–1.77	0.58
Pancreatic cancer				0.58	0.21–1.31	0.17
Objective of therapy						
Adjuvant chemotherapy	0.96	0.69–1.33	0.78	0.77	0.52–1.15	0.20
Grading of adverse event						
Grade 2 + grade 3 vs. grade 1	6.58	3.93–11.02	<0.001	6.25	3.31–11.80	<0.001

## Discussion

The results of this study indicate that assessment of AEs via a telephone consultation service during ambulatory chemotherapy is a useful way to assess the medical condition of outpatients receiving chemotherapy. Notably, assessment of grade ≥2 AEs was significantly associated with changes in clinical schedule (additional call before next appointment to see a doctor, unscheduled visits, and urgent hospitalization due to AEs) and changes in chemotherapy (dosage reduction, deferred treatment, and discontinuation of treatment).

Of the symptoms reported by patients in this study, pain, fever, and nausea/vomiting were the most common. This finding is similar to the common problem of telephone help line in of previous reports [6].

In the ambulatory oncology setting, use of a telephone service can prevent symptoms from becoming unmanageable and possibly help patients avoid unnecessary and costly visits to the hospital. Moreover, such a telephone service may be used to monitor AEs, evaluate the effectiveness of treatment, and increase overall patient satisfaction [7]. However, assessment of AEs using a telephone consultation service differs from the traditional face-to-face assessments by clinicians. Therefore, it is important to monitor AEs using standardized symptom inventory tools administered to patients in an ambulatory care setting during scheduled visits. In this study, we used the CTCAE version 4.0 to assess patient AEs during ambulatory chemotherapy. The assessment of AEs that we found to be significantly associated with the clinical endpoints could add to the current approach to symptom monitoring in ambulatory cancer treatment.

The use of telephone triage to monitor adverse effects is an essential component of contemporary oncology practice. In this study, we showed the potential of telephone triage using CTCAE (grade ≥2 AEs) and its benefit in ambulatory cancer treatment. On the other hand, the triage process is the initial interaction between the patients and the telephone consultants, who must therefore be experienced oncologists and have good communication and assessment skills in order to avoid underestimating the significance of reported symptoms [8].

In this study, we showed a greater benefit of telephone triage for grade 2 but not grade 3 AEs. Grade 3 AEs were generally suspicion of febrile neutropenia (77%). In a telephone consultation service, consultants cannot confirm this AE by blood tests; therefore, consultants with oncology experience are required to adequately assess any suspicions of febrile neutropenia, as guidelines suggest prompt empirical oral antibiotic therapy for low-risk febrile neutropenia patients [9]. In our telephone consultation service, consultants prompted patients to take oral antibiotics if they reported suspicions of febrile neutropenia based on this guideline. This in turn might reduce the risk of unscheduled medical intervention and changes in planned chemotherapy. This study had several limitations. First, it was conducted at an urban tertiary cancer center and was designed as a retrospective cohort study, potentially limiting the generalizability of our findings. CTCAE is widely accepted as the standard classification, however, the classification via telephone is not standard operating procedure. However, this study is the first to report on the use of telephone assessment of AEs during ambulatory chemotherapy using CTCAE. Moreover, we found that this assessment is useful for the triage of patients in addition to predicting severe AEs that could change clinical schedules. Future research should assess that the clinical study of active intervention to prevent severe AEs by use this telephone triage.

## Additional file

Additional file 1:
**Figure S1.** A) Common adverse events reported during consultation. B) Chemotherapy regimens of patients using the telephone consultation service. AC; adriamycin + cyclophosphamide, CBDCA; carboplatin, PTX; paclitaxel, GEM; gemcitabine, CEF; cyclophosphamide + epirubicine + 5-FU, CDDP; cisplatin, BV; bevacizumab.
