# Supplementary figures and images for: Assessment of adverse events via a telephone consultation service for cancer patients receiving ambulatory chemotherapy
Source: BMC Res Notes. 2015 Jul 26;8:315. doi: 10.1186/s13104-015-1292-8 (PMC4514970; doi:10.1186/s13104-015-1292-8)

## Slide 1
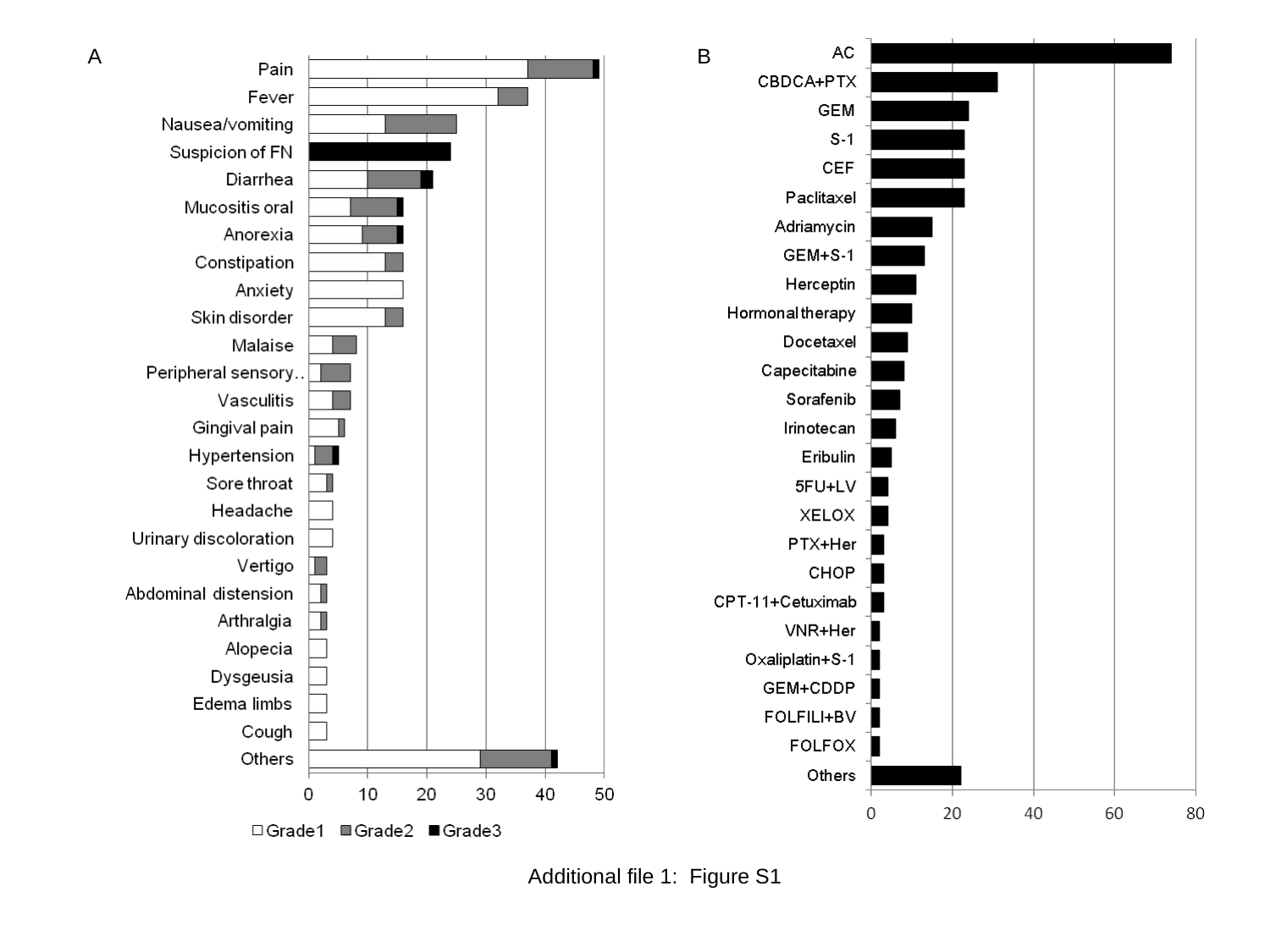

A
B
Additional file 1: Figure S1

Supplement: Additional file 1: — Figure S1. A) Common adverse events reported during consultation. B) Chemotherapy regimens of patients using the telephone consultation service. AC; adriamycin + cyclophosphamide, CBDCA; carboplatin, PTX; paclitaxel, GEM; gemcitabine, CEF; cyclophosphamide + epirubicine + 5-FU, CDDP; cisplatin, BV; bevacizumab. [file 13104_2015_1292_MOESM1_ESM.ppt]
